# *EXOSC3* mutations in pontocerebellar hypoplasia type 1: novel mutations and genotype-phenotype correlations

**DOI:** 10.1186/1750-1172-9-23

**Published:** 2014-02-13

**Authors:** Veerle RC Eggens, Peter G Barth, Jikke-Mien F Niermeijer, Jonathan N Berg, Niklas Darin, Abhijit Dixit, Joel Fluss, Nicola Foulds, Darren Fowler, Tibor Hortobágyi, Thomas Jacques, Mary D King, Periklis Makrythanasis, Adrienn Máté, James AR Nicoll, Declan O’Rourke, Sue Price, Andrew N Williams, Louise Wilson, Mohnish Suri, Laszlo Sztriha, Marit B Dijns-de Wissel, Mia T van Meegen, Fred van Ruissen, Eleonora Aronica, Dirk Troost, Charles BLM Majoie, Henk A Marquering, Bwee Tien Poll-Thé, Frank Baas

**Affiliations:** 1 Department of Genome Analysis, Academic Medical Centre, Amsterdam, the Netherlands; 2 Division of Pediatric Neurology, Emma’s Children’s Hospital, Academic Medical Centre, Amsterdam, the Netherlands; 3 Division of Pathology and Neuroscience, University of Dundee, Dundee, UK; 4 Department of Paediatrics, University of Gothenburg, The Queen Silvia’s Children Hospital, Gothenburg, Sweden; 5 Clinical Genetics, Nottingham City Hospital, Nottingham, UK; 6 Pediatric Neurology, Children’s Hospital, Geneva, Switzerland; 7 Clinical Genetics Service, Southampton University Hospitals Trust, Southampton, UK; 8 Paediatric Pathology, University Hospital Southampton NHS Trust, Southampton, UK; 9 Department of Neuropathology, Institute of Pathology, University of Debrecen, Debrecen, Hungary; 10 Neural Development Unit, UCL Institute of Child Health and the Department of Histopathology, Great Ormond Street Hospital for Children NHS Foundation Trust, London, UK; 11 Paediatric Neurology, Childrens University Hospital, Temple St., Dublin, Ireland; 12 Department of Genetic Medicine and Development, University of Geneva, Geneva, Switzerland; 13 Department of Neurosurgery, University of Szeged, Szeged, Hungary; 14 Clinical and Experimental Sciences, University of Southampton, Southampton, UK; 15 Virtual Academic Unit, Child Development Centre, Northampton, Northants, UK; 16 Clinical Genetics, Great Ormond Street Hospital, London, UK; 17 Department of Paediatrics, University of Szeged, Szeged, Hungary; 18 Department of (Neuro)Pathology, Academic Center, University of the Netherlands, Amsterdam, the Netherlands; 19 Department of Radiology, Academic Medical Center, Amsterdam, the Netherlands; 20 Department of Biomedical Engineering and Physics, AMC, Amsterdam, the Netherlands

**Keywords:** Pontocerebellar hypoplasia, Neurodegeneration, *EXOSC3* gene, Genotype-phenotype correlations

## Abstract

**Background:**

Pontocerebellar hypoplasia (PCH) represents a group of neurodegenerative disorders with prenatal onset. Eight subtypes have been described thus far (PCH1-8) based on clinical and genetic features. Common characteristics include hypoplasia and atrophy of the cerebellum, variable pontine atrophy, and severe mental and motor impairments. PCH1 is distinctly characterized by the combination with degeneration of spinal motor neurons. Recently, mutations in the exosome component 3 gene (*EXOSC3*) have been identified in approximately half of the patients with PCH subtype 1.

**Methods:**

We selected a cohort of 99 PCH patients (90 families) tested negative for mutations in the *TSEN* genes, *RARS2*, *VRK1* and *CASK*. Patients in this cohort were referred with a tentative diagnose PCH type 1, 2, 4, 7 or unclassified PCH. Genetic analysis of the *EXOSC3* gene was performed using Sanger sequencing. Clinical data, MR images and autopsy reports of patients positive for *EXOSC3* mutations were analyzed.

**Results:**

*EXOSC3* mutations were found in twelve families with PCH subtype 1, and were not found in patients with other PCH subtypes. Identified mutations included a large deletion, nonsense and missense mutations. Examination of clinical data reveals a prolonged disease course in patients with a homozygous p.D132A mutation. MRI shows variable pontine hypoplasia in *EXOSC3* mediated PCH, where the pons is largely preserved in patients with a homozygous p.D132A mutation, but attenuated in patients with other mutations. Additionally, bilateral cerebellar cysts were found in patients compound heterozygous for a p.D132A mutation and a nonsense allele.

**Conclusions:**

*EXOSC3* mediated PCH shows clear genotype-phenotype correlations. A homozygous p.D132A mutation leads to PCH with possible survival into early puberty, and preservation of the pons. Compound heterozygosity for a p.D132A mutation and a nonsense or p.Y109N allele, a homozygous p.G31A mutation or a p.G135E mutation causes a more rapidly progressive course leading to death in infancy and attenuation of the ventral pons.

Our findings imply a clear correlation between genetic mutation and clinical outcome in *EXOSC3* mediated PCH, including variable involvement of the pons.

## Background

Pontocerebellar hypoplasias represent a group of autosomal recessive neurodegenerative disorders with prenatal onset. Patients of all subtypes show variable hypoplasia/atrophy of pons and cerebellum and severe motor and cognitive impairments. Based on clinical and genetic criteria, eight subtypes of PCH have been classified (PCH1-8). PCH1 (MIM607596, 614678) is a distinctive subtype of PCH, and is characterized by degeneration of motor neurons in the anterior spinal horn, morphologically similar to spinal muscular atrophy (SMA). PCH1 patients present a broad phenotypic spectrum, ranging from neonatal death [1] to survival into puberty ([2] and patients 7-I and 7-II in this paper). In few families diagnosed with PCH1, mutations have been found in *TSEN54*[3], *RARS2*[4] and vaccinia-related kinase 1 (*VRK1*) [5]. Approximately half of PCH1 families carry mutations in *EXOSC3*, the gene encoding exosome component 3 [2,6-9].

In this study, we report *EXOSC3* mutations in twelve families with PCH1. We categorised the patients according to genetic mutation, and correlate this with clinical severity and size of the ventral pons.

## Methods

### Patient cohort

Our laboratory is a reference centre for PCH genetic analysis. We received EDTA-blood or DNA samples from hospitals and institutes worldwide for genetic analysis of genes associated with PCH. For this study, we selected DNA material from a cohort of 99 patients (90 families) diagnosed by referring specialists with PCH type 1, 2, 4, 7 or an unknown PCH-like anomaly. All cases were negative for mutations in genes known to cause PCH (*TSEN54*, *TSEN34*, *TSEN2*, *RARS2*, *VRK1* and *CASK*). Informed consent was obtained by referring specialists.

### Genetic analysis

PCR primer pairs for *EXOSC3* [NM_016042.2] were designed for all exons including intron-exon boundaries using Primer3 software (http://frodo.wi.mit.edu/). For primer sequences, see Additional file 1. Sanger sequencing of PCR amplified DNA was performed using BigDyeTerminator chemistry (Applied Biosystems) and analysed on an ABI3730xl sequencer. Sequences were analysed using CodonCode Aligner software 3.6.1. Analysis of gene mutations was done with the Alamut software package (Interactive Biosoftware, version 2.0), which includes the splice site prediction algorithms SpliceSiteFinder, MaxEntScan and Human Splicing Finder. Detection of large deletions in *EXOSC3* was initially performed with you-MAQ assay (Multiplicon), using primers in the 5’UTR and intron 3 (see Additional file 1). Detailed analysis of the deletion in patient 8 was performed with the SequalPrep Long PCR Kit (Life Technologies), using primers in the 3’UTR and upstream of *EXOSC3* (see Additional file 1).

### Neuroimaging

Routine MR images of *EXOSC3* mutation positive patients were re-examined. Coronal cerebellar images from patients found to be positive for mutations in *EXOSC3* were subclassified as previously described [4]. The main impact of the process that causes pontine hypoplasia/atrophy is on the ventral pons, which leaves the dorsal area (tegmentum) relatively unaffected [10,11]. The ventral pons and tegmentum can be distinguished on routine MRI. On midsagittal MR scans, surfaces of these areas were determined using ITK-SNAP 2.4.0 software [12] and ventral pons/(ventral pons+tegmentum) (VP/(VP+T)) ratios were determined as a measure for pontine hypoplasia/atrophy. Three-dimensional images were constructed based on a series of MR scans using the same software. The control group consists of MR images obtained from children (n=23; neonatal to 11y) referred by paediatric neurologists for diagnostic MRI, whose brain MR image was considered normal.

### Histological stainings

Paraffinised sections of the pons were stained with Luxol/PAS following standard protocols as described previously [13].

## Results

### EXOSC3 *molecular analysis*

Screening of a cohort of 99 patients (90 families) with various PCH subtypes revealed *EXOSC3* mutations in fourteen PCH patients (twelve families, Table 1). Six patients were homozygous for the c. 92G>C (p.G31A) mutation, three patients were homozygous for the c.395A>C (p.D132A) mutation, and one patient was homozygous for the c.404G>A (p.G135E) mutation. We found four patients to be compound heterozygous for *EXOSC3* mutations (patients 8, 9, 10 and 11). Patient 8 had a hemizygous p.D132A mutation and a heterozygous 6,171 nucleotide large deletion containing the promoter region, the first three exons and a part of intron 3 of the *EXOSC3* gene (g.del37781240-37787410), without affecting adjacent genes. Patient 9 had a heterozygous p.D132A mutation and a c.743_749delinsA mutation, leading to a premature stop codon (p.L248*). Patient 10 had a p.D132A mutation on the maternal allele. On the paternal allele, this patient had a c.334G>A (p.V112I) variation in addition to a nine nucleotide duplication leading to a premature stop codon (c.325-4_329dupGTAGTATGT; p.P111*). The p.V112I amino acid change was predicted to be benign by SIFT (score 0,32) and Align GVGD (class C0), and possibly damaging by PolyPhen-2 (score 0,949). Patient 11 had the p.D132A mutation plus a c.325T>A (p.Y109N) mutation. The c.325 residue is the first nucleotide of exon 2, but the mutation is not predicted to affect splicing by the SpliceSiteFinder, MaxEntScan and Human Splicing Finder software. Unfortunately, tissue was not available for splicing studies. The p.Y109N missense mutation was predicted to be deleterious by PolyPhen-2 (score 1,000), SIFT (score 0,0) and Align-GVGD (class C65). In all cases, we confirmed the mutations by sequencing the parents. In case of expected compound heterozygosity, we could show that the mutations were on different alleles.

**Table 1:** **Clinical data of 14 patients with *EXOSC3 *** **mutation**

	***1***	***2***	***3***	***4***	***5-I***	***5-II***	***6***	***7-I***	***7-II***	***8***	***9***	***10***	***11***	***12***
**Nucleotide change**	c.92G > C	c.92G > C	c.92G > C	c.92G > C	c.92G > C	c.92G > C	c.395A > C	c.395A > C	c.395A > C	c.395A > C (he) g.del37781240-37787410 (he)	c.395A > C (he) c.743_749delinsA (he)	c.325-4_329dupGTAGTATGT (he) c.334G > A (he) c.395A > C (he)	c.325 T > A (he) c.395A > C (he)	c.404G > A
**Amino acid change**	p.G31A	p.G31A	p.G31A	p.G31A	p.G31A	p.G31A	p.D132A	p.D132A	p.D132A	p.D132A; deletion exon 1-3	p.D132A; p.L248*	p.P111*; p.V112I; p.D132A	p.Y109N; p.D132A	p.G135E
**Ethnic background**	Roma	Roma	Roma	Roma	Roma	Roma	Caucasian	Caucasian	Caucasian	Caucasian	Caucasian	Caucasian	Caucasian	Pakistan
**Pregnancy duration**	39w, CS	at term	38w	37w	37w	40w	39w	u	35w	39w	38w	42w	41w	40w
**Hypotonia at birth**	+	+	+	+	+	+	+	+	+	+	+	±	+	+
**OFC (SD)**^**a **^**(age)**	−4 (1.5 m)	−2.5 (birth)	0 (birth)	+3 (4.5mo)	0 (4mo)	+2.5 (4mo)	+3 (4.5mo)	−0.5 (11y)	−2 (6.5y)	−1 (birth)	u	−0.5 (10w)	−1.5 (6.5mo)	−1 (8w)
**Nystagmus**	-	-	u	+	-	-	+	+	+	-	u	-	-	+
**Optic atrophy**	Pale optic disc	-	u	-	-	-	-	u	u	+	u	-	Small optic discs	Pale optic disc
**Seizures**	-	-	-	-	-	-	-	+	-	+ West syndrome at 5 mo	-	-	-	-
**Dyskinesia/dystonia**	-	-	-	-	-	-	+ 1 episode, admitted with high temp and pneunomia	+	+	-	-	-	-	-
**Tendon reflexes**	absent	absent	absent	absent	absent	absent	brisk	brisk	reduced	reduced	absent	reduced	absent	absent
**Response on visual/auditory stimuli**	-	-	u	-	-	-	++	+	+	±	-	±	-	-
**Age at death (cause)**	4.5mo (cardiac arrest)	7mo (pneumonia, sepsis)	5d (respiratory failure)	5mo (u)	6mo (viral infection)	4mo (u)	7y (respiratory failure)	12y (GI failure)	10y (pseudomonas infection)	6mo (respiratory infection)	14w (respiratory failure)	6mo (respiratory infection)	8.5mo (respiratory failure)	8w (respiratory failure)
**Lower motor neuron signs**	Neurogenic muscle atrophy	Neurogenic muscle atrophy	u, diagnosed following patient 5-II (cousin)	Tongue fasciculations, denervation (EMG), neurogenic muscle atrophy	u, diagnosed following patient 5-II (sister)	Muscle denervation (EMG)	u	u, diagnosed following patient 7-II (brother)	Denervation (EMG)	Neurogenic muscle atrophy	Denervation (EMG)	Denervation (EMG), reduced motor nerve conduction velocity	Tongue fasciculations, neurogenic muscle atrophy	Denervation, neurogenic muscle atrophy

he = heterozygous; d = days; w = weeks; mo = months; y = years; u = unknown; ++ = markedly present; + = present; ± = mildly present; - = not present.

^a^SD for head circumference according to WHO standards (http://www.who.int/childgrowth/standards/hc_for_age/en/index.html).

In our cohort with different PCH subtypes, all twelve families harbouring *EXOSC3* mutations were diagnosed as PCH1. In four other PCH1 families in our cohort we could not detect mutations in coding regions of this gene. No *EXOSC3* mutations were found in patients diagnosed with other types of PCH. Therefore, *EXOSC3* mutations seem to be exclusively associated with the PCH1 subtype. The patients harbouring the p.G31A mutation are all of Romani descent - although living in different countries (Sweden and Hungary) - suggesting a common founder as proposed earlier [7,9]. The families with the p.D132A allele are all of Caucasian descent.

### *Clinical data of patients with* EXOSC3 *mutations*

Most *EXOSC3* mutation positive patients were previously diagnosed as PCH1 based on clinical features. Although patients 3 and 5-I were not tested for lower motor neuron symptoms, they were diagnosed following the diagnosis of patient 5-II, which is patient 5-IIs sister and patient 3s cousin. Interestingly, patients 6, 7-I and 7-II were initially not diagnosed as PCH1, as it was yet unknown that the PCH1 spectrum was broad, and that patients could survive childhood [6]. The finding of depleted motor neurons on autopsy in these patients confirmed PCH1 diagnosis.

Clinical features of *EXOSC3* positive patients in our cohort are summarized in Table 1. Based on genetic mutations, our cohort can be divided in four subgroups: 1. homozygosity for the p.G31A mutation; 2. homozygosity for the p.D132A mutation; 3. compound heterozygosity for the p.D132A mutation on one allele and a deletion, nonsense or missense mutation on the other allele; 4. homozygosity for the p.G135E mutation.

In the p.G31A group (six patients, five families), hypotonic pareses were present at birth in five patients, and observed at one month in one patient. No patients were born with polyhydramnios, though all suffered from swallowing insufficiency. Tendon reflexes and responses to external stimuli were absent in all patients in this group. At birth, severe contractures were present in two patients: patient 2 presented with contractures in the knee joints and had equinovarus foot deformities; patient 4 had contractures in hips, ankles, elbows, hands and fingers. Dyskinesia or seizures were not observed in this group. Mild facial dysmorphisms were observed in three of the six patients, including low set ears (2/6), broad nasal bridge (2/6) and short palpebral fissures (2/6). Two patients had microcephaly (patient 1: -4 SD at 1.5 months of age; patient 2: -2.5SD at birth), while others had normal or even large OFCs (patient 4: +3SD at 4.5 months of age) at the time of presentation. All homozygous p.G31A patients died during infancy (median age of death 4.75 months, range 5 days to 7 months).

In the p.D132A group (three patients, two families), hypotonia was found at birth in two patients, and at 13 weeks in one patient. No polyhydramnios was observed in this group. Tendon reflexes were either brisk (2/3 patients) or reduced (1/3 patients). Patients in this group were able to respond to visual and auditory stimuli. For example, patient 6 recognized familiar faces and voices, made social eye contact and babbled at the age of six years. Patients 7-I and 7-II had remarkably good cognitive skills compared to other PCH patients - the boys were able to move a computer mouse to make simple ‘yes’ or ‘no’ choices in a computer game. Due to severe motor impairments adequate testing of IQ was not possible, but parents and caretakers considered these brothers’ intellectual skills age appropriate. Swallowing insufficiency was present in all three patients. Severe contractures at birth were not seen in this group. From the age of 5 and 4 years respectively, patients 7-I and 7-II developed progressively internally rotated arms and weak, claw-like deformity of the hands. Dyskinesia was reported sporadically in patient 6 and regularly in patients 7-I and 7-II. Patient 7-I had initially few generalised seizures per year, which increased in frequency and severity until death. This patient also developed intense visceral pains and ultimately succumbed to his gastrointestinal tract malfunctioning. All three patients in this group survived into childhood (age of death 7, 12 and 10 years respectively).

The third group consists of patients with a p.D132A mutation in combination with a null allele or missense mutation in the *EXOSC3* gene (four patients, four families). All patients were hypotonic at birth, with multiple contractures in three patients. Two patients could be breastfed or bottle fed in the first weeks of life, but developed swallowing difficulties later on. Tendon reflexes were absent (2/4) or reduced (2/4). Dyskinesia was not reported in this group. Seizures are reported in two patients: patient 8 developed West Syndrome at the age of 5 months and in patient 10 seizures were reported by his parents in the days before his death. Facial dysmorphisms in two patients included low set ears and large soft ear helices. In addition, patient 10 presented fat distribution as seen in CDG1a, and patient 11 had a small penis and hypoplastic scrotum. The patients died during infancy (median age of death 6 months, range 3.5 to 8.5 months).

One patient in our cohort was homozygous for the p.G135E (c.404G>A) mutation. Born from consanguineous parents, he presented reduced fetal movements. At birth, he was hypotonic and had contractures of elbows and knees. He had nystagmus, pale optic discs and failed to respond to external stimuli. The patient died at the age of 8 weeks.

### Brain imaging studies

**Figure 1:**
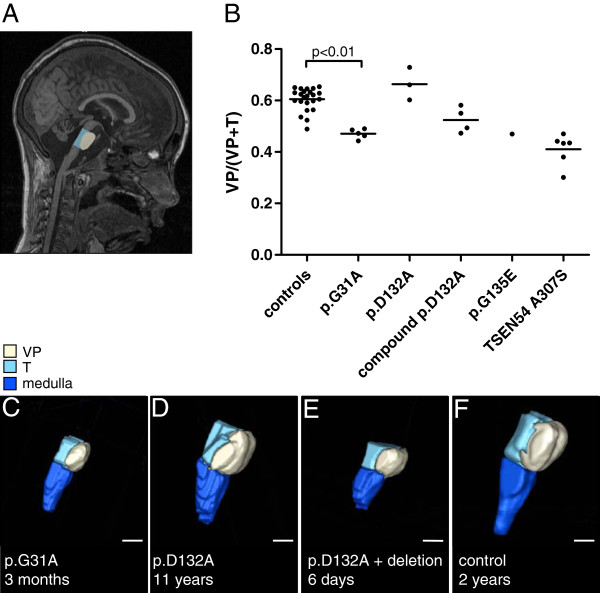
**Ventral pons/tegmentum ratios in PCH patients.** On midsagittal MR images, surfaces of ventral pons and pontine tegmentum were defined as shown in Figure **A**. Patients with a homozygous p.D132A mutation in *EXOSC3* (n=3) present a pons/tegmentum ratio comparable to controls (n=23, age neonatal to 11y). Patients with a homozygous p.G31A mutation (n=5), a p.D132A mutation plus a nonsense or p.Y109N allele (n=4) or a homozygous p.G135E mutation (n=1) show a decreased ratio, approaching that seen in patients with a p.A307S mutation in the *TSEN54* gene (n=6) **(B)**. Three-dimensional images were constructed of the pons, tegmentum and part of the medulla. The reconstructions show an attenuated pons in a patient 5-I (**C**, homozygous p.G31A mutation) and patient 8 (**E**, p.D132A plus large deletion) compared to patient 7-I (**D**, homozygous p.D132A) or a control subject **(F)**. Scale bar in C-F=1cm. VP=ventral pons; T=tegmentum.

MR images of the brain were available for all patients, except for patient 1. Based on coronal images, we categorised the cerebellar hemisphere patterns as described previously [4]: a dragonfly type (flattened hemispheres, relative prominence of vermis), a butterfly type (small but normally proportioned cerebellum) and a postnatal atrophy-like type (cerebellar atrophy rather than hypoplasia with the hemispheres reaching the margins of the posterior fossa in at least one spot). As a measure for pontine hypoplasia/atrophy we determined the VP/(VP+T) ratio on midsagittal MR images (Figure 1A).

**Figure 2:**
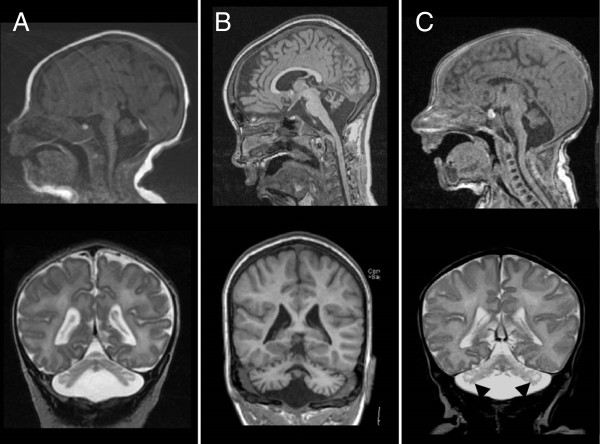
**Brain MRI of PCH patients with an *EXOSC3 *mutation.** Sagittal and coronal images of a patient with a homozygous p.G31A mutation (**A**, patient 5-II, age 2w), a patient with a homozygous p.D132A mutation (**B**, patient 7-I, age 11y) and a patient with a p.D132A mutation and large deletion (**C**, patient 8, age 1mo). Cerebellar cysts in this last patient are indicated by arrow heads.

In the p.G31A group, four of the five cerebellar hemisphere pathologies resembled a dragonfly pattern (Figure 2A). Patient 5-I presented a butterfly-like cerebellar pattern, albeit hemispheres are smaller than a typical butterfly pattern. In the p.G31A group, the pons is significantly attenuated compared to controls (p<0.01; Figure 1B, C). Supratentorial abnormalities are seen in three of the five patients in this group: these patients had widened extracerebral CSF spaces, and one patient (patient 4, born at 37 weeks gestational age) presented delayed neocortical maturation.

All three patients with a homozygous p.D132A mutation showed a postnatal atrophy-like cerebellar pattern (Figure 2B). Patient 6 showed supratentorial abnormalities: she had enlarged ventricles due to atrophy of the basal ganglia. Patient 7-II underwent a CT scan at the age of one year, which was normal. An MRI performed at the age of five years showed a small cerebellum, suggesting progressive atrophy. The VP/(VP+T) ratio in this group equals that of healthy controls (Figure 1B, F), indicating a largely unaffected pons in patients with a p.D132A mutation (Figure 1B, D).

Three of the four patients in the compound heterozygous group presented a dragonfly cerebellum. One patient in this group (patient 9) presented a butterfly pattern. Cerebellar cysts were common in this group (3/4 patients) (Figure 2C). Supratentorial abnormalities are seen in patient 10 (mild atrophy of the cerebral hemispheres and caudate nuclei and widened ventricles) and patient 11 (thin corpus callosum). Midsagittal pons measurements showed pontine attenuation in this group (Figure 1B, E).

The patient homozygous for the p.G135E mutation presents with cerebellar cysts and no supratentorial abnormalities. Since no coronal image was available for this patient, it is hard to describe the pattern type of the cerebellar hemispheres. Ratio of midsagittal pons/tegmentum surface in this patient is comparable to the p.G31A group (Figure 1B).

To conclude, the genotype-phenotype correlation of the various *EXOSC3* mutations is reflected in the severity of cerebellar and pontine hypoplasia.

### Neuropathological findings

**Figure 3:**
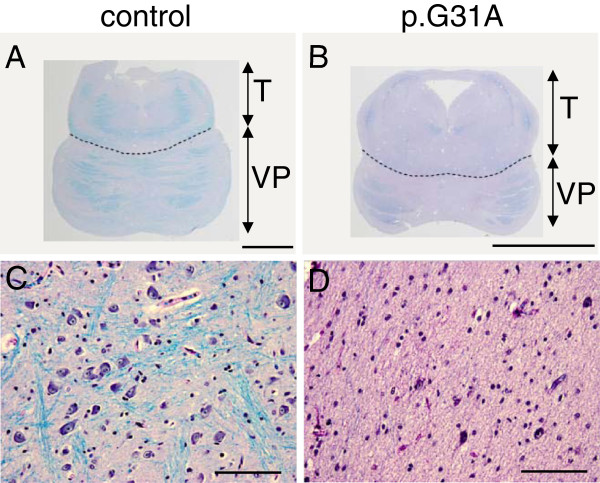
**Pons abnormalities in patient with p.G31A mutation.** Luxol/PAS staining of the pons shows small pons and reduction of transverse pontine fibres **(B)** and pontine neurons **(D)** in a patient with a homozygous p.G31A mutation (patient 1) compared to age matched control **(A and C)**. Scale bar **A** and **B**=0.5cm, **C** and **D**=100μm. VP=ventral pons; T=tegmentum.

Autopsy was performed on one patient with a p.G31A mutation (patient 1). No MRI was made of this patient, but post mortem results showed atrophy of cerebellar hemispheres, inferior and middle cerebellar peduncles. The cerebellar hemispheres were poorly developed and presented a smooth surface. Histological analysis showed a reduced number of Purkinje cells and a fragmented dentate nucleus. The number of transverse pontine fibres and pontine neurons was reduced compared to control tissue (Figure 3).

Post mortem studies were performed on all three patients with a p.D132A mutation. In all, atrophy of cerebellar hemispheres and vermis was noted, including a reduced number of Purkinje cells. Characteristic for PCH1 – the number of motor neurons in the anterior spinal horn was reduced. Residual motor neurons showed a shrunken and angulated morphology. In one patient (7-I), depletion of neurons in the dorsal horn was also seen. Examination of the pons revealed proportionate reduction of transverse pontine fibres, descending fibres and pontine nuclei in patient 6. No pontine abnormalities were found in patient 7-I and 7-II, in line with the normal appearance of the pons on MRI. Cerebral anomalies were found in patients 7-I and 7-II: both presented neuron loss, gliosis and calcifications in the thalamus.

## Discussion

We performed genetic screening on 99 subjects with various types of PCH. Among this cohort, we identified fourteen PCH1 patients (twelve families) with mutations in *EXOSC3*, suggesting that mutations in this gene are restricted to PCH subtype 1. Four families with PCH1 that we screened for *EXOSC3* mutations did not have mutations in coding regions of this gene. In addition to the *EXOSC3* mutations described previously, we identified five novel variants in *EXOSC3*, leading to an amino acid substitution or a nonsense allele.

After the identification of *EXOSC3* as a PCH associated gene [6], many different mutations have been found in this gene (Table 2). This genetic heterogeneity is reflected in the broad phenotypic spectrum of *EXOSC3* mediated PCH, ranging from relatively mild (e.g. compound heterozygous p.D132A/p.V80F mutation [2]) to severe (e.g. homozygous p.G31A mutation [9]). Findings by us and others [6-8], show that *EXOSC3* mutations underlie about half of the PCH1 cases. *EXOSC3* mediated PCH shows a genotype-phenotype correlation: the p.D132A mutation leads to a relatively prolonged disease course including survival into childhood, limited social interaction and preservation of the pons. A homozygous p.G31A mutation, or a p.D132A in combination with a nonsense or p.Y109N mutation leads to a more severe type of PCH reflected in clinical symptoms, death during infancy and hypoplasia of the pons. The patient with a homozygous p.G135E mutation shows a similarly severe phenotype.

**Table 2:** **Overview of mutations identified in *EXOSC3***

**Allele A**	**Allele B**	**Reference**
p.G31A (c.92G > C)	p.G31A (c.92G > C)	[6,7,9] This paper
p.G31A (c.92G > C)	p.W238R (c.712 T > C)	[6,7]
p.D132A (c.395A > C)	p.D132A (c.395A > C)	[6-8] This paper
p.D132A (c.395A > C)	start codon affected (c.2 T > C)	[7]
p.D132A (c.395A > C)	p.P52Rfs*2 (c.155delC)	[7]
p.D132A (c.395A > C)	p.D76Gfs*49 (c.226dupG)	[7]
p.D132A (c.395A > C)	p.V80F (c.238G > T)	[2]
p.D132A (c.395A > C)	p.V99Wfs*11 (c.294_303del)	[6]
p.D132A (c.395A > C)	p.Y109N (c.325 T > A)	This paper
p.D132A (c.395A > C)	p.P111*; p.V112I (c.325-4_329dupGTAGTATGT; c. 334G > A)	This paper
p.D132A (c.395A > C)	p.A139P (c.415G > C)	[6]
p.D132A (c.395A > C)	exon 3 skipping (c.475-12A > G)	[6,7]
p.D132A (c.395A > C)	p.C184Lfs*19 (c.551delG)	[7]
p.D132A (c.395A > C)	p.L248* (c.743_749delinsA)	This paper
p.D132A (c.395A > C)	deletion exon 1–3 (g.del37781240-37787410)	This paper
p.G135E (c.404G > A)	p.G135E (c.404G > A)	This paper

Previous research suggested that patients with an *EXOSC3* mutation show a relatively preserved pons in comparison to other types of PCH [4,7,8]. We have quantified this and we demonstrate that the pons/tegmentum ratio on midsagittal MR images of patients with a homozygous p.D132A mutation indeed resembles that of healthy controls. However, in patients with other mutations in *EXOSC3*, the pons/tegmentum ratio was decreased. The ratio in patients with a homozygous p.G31A mutation does not differ significantly from PCH patients with a p.A307S mutation in the *TSEN54* gene, although we should keep in mind that the cohorts are small (p.G31A five patients; p.A307S six patients). Interestingly, our control group shows that the ventral pons/tegmentum ratio is rather stable over a large age range (0 to 11 years). MRI analysis showed the presence of cerebellar cysts in 4/14 patients with mutations in *EXOSC3*. Intracerebellar cysts have been reported before in few severe cases of PCH1 [3,14]. Their morphology closely resembles cysts in PCH2 [4]. Our results support the occurrence of cysts in severe PCH1.

Due to motor handicaps intellectual performance could not be studied in detail, but behavioral study suggests normal cognition in some patients in the p.D132A group ([2], patients 7-I and 7-II in this paper).

The visceral pain of which patient 7-I suffered from, is a phenomenon not previously reported in PCH1. Neuropathological post-mortem examination of this patient revealed degeneration of neurons in both the ventral and dorsal spinal horn. Degeneration of neurons in the dorsal horn may account for the chronic visceral pains in this patient. Patient 7-II had a normal CT brain scan at 1 year of age, but cerebellar hypoplasia on MRI at 5 years of age, demonstrating the progressive nature of the disease. Additionally, it emphasizes the importance of serial brain imaging in diagnosing PCH.

Motor disorders such as dyskinesia and spasticity were only observed in the p.D132A group which is charactarised by prolonged survival.

To conclude, we show that within the broad spectrum of *EXOSC3* mediated PCH, clear genotype-phenotype correlations can be made. A homozygous p.D132A mutation leads to a more chronic form of PCH, with survival into childhood, and preservation of the pons. Compound heterozygosity for a p.D132A mutation and a nonsense or p.Y109N allele, a homozygous p.G31A mutation or a p.G135E mutation causes a severe disease course including death during infancy and hypoplasia of the pons.

## Conclusions

We identified new nonsense and missense mutations in the *EXOSC3* gene and we show that mutations in this gene are exclusively found in PCH1 patients. There are evident genotype-phenotype correlations in *EXOSC3*-mediated PCH reflected in clinical outcome, age of death and pons hypoplasia: patients with a homozygous p.D132A mutation have a prolonged disease course compared to patients with a p.D132A allele plus a nonsense or p.Y109N mutation. Patients with a homozygous p.G31A mutation and the patient with a homozygous p.G135E mutation present a similarly severe phenotype with death in infancy. Our results refine the current view of an unaffected pons in EXOSC3 mediated PCH.

## Abbreviations

PCH: Pontocerebellar hypoplasia; SMA: Spinal muscular atrophy; PCR: Polymerase chain reaction; MR(I): Magnetic resonance (imaging); VP: Ventral pons; T: Tegmentum.

## Competing interests

The authors declare to have no competing interests.

## Authors’ contribution

VRCE collected and analysed the data and wrote the manuscript. PGB had a valuable contribution in writing and revising the manuscript. FvR, MBD-dW and MTvM performed molecular genetic analysis. PGB, JFN and BTP-T analysed MRI scans and were closely involved in discussions. JNB, ND, AD, JF, NF, DF, TH, TJ, MK, PM, AM, JARN, DO’R, SP, ANW, LW, MS and LS provided clinical information. EA, DT, TJ and TH provided post mortem tissue and stainings. CBM and HAM were involved in neuroradiological analysis. FB coordinated the study and the writing of the manuscript. All authors read, revised and approved the final manuscript.

## Supplementary Material

Additional file 1 Supplementary methods. Click here for file
