# Supplementary material for: EXOSC3 mutations in pontocerebellar hypoplasia type 1: novel mutations and genotype-phenotype correlations
Source: Orphanet J Rare Dis. 2014 Feb 13;9:23. doi: 10.1186/1750-1172-9-23 (PMC3928094; doi:10.1186/1750-1172-9-23)
Supplement: Additional file 1 — Supplementary methods. [file 1750-1172-9-23-S1.doc]

**Supplementary methods**

- Coding regions an intron-exon boundaries of the EXOSC3 gene were PCR amplified using the primers listed below. Every primer was tagged at the 5’ end with a M13 sequence (forward: 5’-TGTAAAACGACGGCCAGT-3’; reverse: 5’-CAGGAAACAGCTATGACC-3’).

| **Exon** | **Forward primer** | **Reverse primer** |
| --- | --- | --- |
| **1** | 5’-GGAAACGGAAAGTCCTCAAG-3’ | 5’-GGGTCCCTCTCTTCTTTTGG-3’ |
| **2** | 5’-CACAAAGGACTTGCTGAAGG-3’ | 5’-GCCTTCTGGATATGTGAGTGTTC-3’ |
| **3** | 5’-CAGTCAGTCCCTTAGCCACAG-3’ | 5’-TGGATACTGATTTCTAACTCTGAGGA-3’ |
| **4** | 5’-CGTGCCTTGTTCTTGTTGAA-3’ | 5’-ACAAAAGCGTGGGTGAAAAC-3’ |

- Primers used for the you-MAQ assay are listed below. Genomic positions relative to hg19 are given between brackets.

| **Amplicon** | **Forward primer** | **Reverse primer** |
| --- | --- | --- |
| FlagMAQ_  EXOSC3.1 | 5'AGCGGATAACAATTTCACACAGGAAATCACAGAGGTGGGCTATG-3'  (37,781,063 – 37,781,084) | 5'-GTTTCTTAACCAAGCACCACCCTTATG-3'  (37,781,306 – 37,781,285) |
| FlagMAQ_  EXOSC3.2 | 5'AGCGGATAACAATTTCACACAGGCTTTGGTGTGTATTTCTCCCTAGAC-3'  (37,786,001 – 37,786,025) | 5'-GTTTCTTGGCCATGTAGGGCTATCG-3' (37,786,080 – 37,786,063) |

- Primers used for PCR amplification and sequencing for detailed analysis of the large deletion in patient 8 are:

Upstream *EXOSC3*: 5’-TTTTTGCCTCAACTGACCTG-3’

3’ UTR of *EXOSC3*: 5’-AGCTAAAGTGGCCTTGGTGA-3’
